# A 1.5-Year Longitudinal Study of Social Activity in Patients With Schizophrenia

**DOI:** 10.3389/fpsyt.2019.00567

**Published:** 2019-08-09

**Authors:** Kazutaka Ohi, Chika Sumiyoshi, Haruo Fujino, Yuka Yasuda, Hidenaga Yamamori, Michiko Fujimoto, Tomiki Sumiyoshi, Ryota Hashimoto

**Affiliations:** ^1^Department of Neuropsychiatry, Kanazawa Medical University, Uchinada, Japan; ^2^Medical Research Institute, Kanazawa Medical University, Uchinada, Japan; ^3^Faculty of Human Development and Culture, Fukushima University, Fukushima, Japan; ^4^Graduate School of Education, Oita University, Oita, Japan; ^5^Department of Psychiatry, Osaka University Graduate School of Medicine, Suita, Japan; ^6^Department of Clinical Epidemiology, Translational Medical Center, National Center of Neurology and Psychiatry, Kodaira, Japan; ^7^Department of Preventive Intervention for Psychiatric Disorders, National Institute of Mental Health, National Center of Neurology and Psychiatry, Kodaira, Japan; ^8^Molecular Research Center for Children’s Mental Development, United Graduate School of Child Development, Osaka University, Suita, Japan; ^9^Department of Pathology of Mental Diseases, National Institute of Mental Health, National Center of Neurology and Psychiatry, Kodaira, Japan

**Keywords:** schizophrenia, social activity, social function, daily living skills, IQ, longitudinal study

## Abstract

Patients with schizophrenia exhibit impairments in their social activity, intelligence quotient (IQ), daily living skills, and social function. Social activity is a high-order outcome measure of their lives. Here we attempted to longitudinally evaluate the effects of IQ, daily living skills, social function, psychiatric symptoms, and medications on social activity in patients with schizophrenia. The purpose of the current study is to identify the specific factor that affects longitudinal changes in social activity. Sixty-five patients with schizophrenia were assessed at two time points [time 2 (T2, follow-up) − time 1 (T1, baseline) = 1.71 ± 0.79 years]. Social activity, IQ, daily living skills, and social function were assessed using the Social Activity Assessment (SAA; h/week), short form of the Wechsler Adult Intelligence Scale (WAIS)-III (WAIS-SF), University of California San Diego (UCSD) Performance-Based Skills Assessment (UPSA), and Social Functioning Scale (SFS), respectively. IQ, daily living skills, social function, and social activity were significantly improved between T1 and T2 (*t* = 2.0–4.4, *p* = 0.048–3.60 × 10^−5^). IQ, daily living skills, and social function positively correlated with social activity (lowest *p* = 1.27 × 10^−5^), and psychiatric symptoms negatively correlated with social activity over time (lowest p = 3.26 × 10^−9^). The longitudinal change in social activity was independently and positively correlated with a change in social function (*beta* = 0.35, *p* = 4.63 × 10^−3^), particularly interpersonal communication (*beta* = 0.35, *p* = 4.32 × 10^−3^). The longitudinal changes in other factors did not directly affect the change in social activity (*p* > 0.05). Based on these findings, social activity is more affected by social function than by other factors.

## Introduction

Social and occupational impairments are a long-recognized core feature of patients with first-episode and chronic schizophrenia (1, 2). These impairments are linked to a wide range of factors, including symptoms such as delusions, hallucinations, blunted affect and withdrawal, cognitive impairments, decreased functional capacity, and real-life dysfunction, in patients with schizophrenia (1–4). Cognitive function contributes to work skills and is mediated by functional capacity (5, 6). Additionally, social skills and functional capacity are related to social, occupational, and real-world functional outcomes (7–10). Because schizophrenia ranks among the first 20 leading causes of disability worldwide (11), despite the advances in pharmacological treatments for psychiatric symptoms, long-term treatment for deficits in social and occupational functioning may be required.

The alleviation of psychiatric symptoms contributes to improvements in real-life functioning but is not sufficient to attain recovery from the disorder (12). Although the definition of recovery varies among studies, recovery does not equate to symptomatic remission (13, 14). Functional recovery is defined as when symptoms are mild and stable enough to not interfere with normal functioning in social activities and relationships (15). Although there is no consensus on predictors of recovery (15, 16), experts agree that both negative symptoms and cognitive function are considered to cause significant impact on functional recovery without superiority (15). Furthermore, functional improvements including independent living, employment, relationships, and social contacts are considered essential for not only symptomatic remission but also recovery (12, 16, 17). Patients with schizophrenia spend less time on work, housework/childcare, studying, and social and leisure activities compared to the general population (1, 18). In addition, some patients with schizophrenia do not seek work. Reducing inactivity and increasing the time spent engaging in productive tasks have been shown to be valuable indicators of recovery (1, 19). Social activity is calculated as the sum of work hours per week (h/week) of work for pay, housework, and/or studying. Social function is assessed as activity frequency in patients with schizophrenia using the Social Functioning Scale (SFS) (20), while the SFS does not capture the time spent in weekly social activity. In contrast, social activity is assessed as the time spent (h/week) at a place of employment, on housework, or studying using the Social Activity Assessment (SAA). Therefore, social activity, i.e., work hours per week (h/week), may be a useful measure of further treatment evaluations or clinical assessments of recovery.

Although social activity is influenced by illness severity, cognitive functions, daily living skills, and social function, the identification of the most appropriate indices to capture the common factor associated with these impairments is not an easy task. Although several studies have investigated longitudinal trajectories of social and cognitive functions in patients with schizophrenia, these results were inconsistent (21–27). Some components of these impairments (e.g., ability to follow rules at school and/or work and cognitive functions) appear relatively stable over time before and/or after the onset of schizophrenia (21–24, 27), while other components (e.g., ability to make and maintain friends, social and cognitive functions), and a subgroup of the patients, showed a progressive decline or improvement with increased illness chronicity (21, 22, 25, 26). Additionally, impairments in premorbid adjustments and cognitive and community functions have been reported in patients with schizophrenia and their unaffected relatives (21, 23, 28–30), suggesting that these impairments may be due to shared genetic and/or environmental risk factors. To date, little is known about the longitudinal effects of cognitive function, daily living skills, social function, psychiatric symptoms, and medications on social activity (h/week) in patients with schizophrenia. If social activity, a useful measure of recovery, would be prospectively as well as cross-sectionally affected by intellectual quotient (IQ), daily living skills, social function, psychiatric symptoms, and/or medications, the identification of these relationships would be helpful for clinical trials and intervention studies for recovery in patients with schizophrenia. We hypothesized that the longitudinal changes in social activity would be affected by the longitudinal changes of the IQ, daily living skills, social function, psychiatric symptoms, and/or medications.

In this study, we performed a 1.5-year evaluation of the longitudinal effects of the IQ, daily living skills, social function, psychiatric symptoms, and medications on social activity in patients with schizophrenia using assessments conducted at two time points. The main purpose of this study was to identify a specific factor affecting the longitudinal changes in social activity.

## Methods

### Participants

Sixty-five patients with schizophrenia were included in this study [44.6% males (29 males and 36 females); mean age ± SD, 33.7 ± 12.8 years at baseline (time 1, T1), 35.5 ± 13.0 years at follow-up (time 2, T2)]. All patients were biologically unrelated within the second degree of relationship and were of Japanese descent (31–35). Patients with schizophrenia were recruited from both the outpatient and inpatient populations at Osaka University Hospital. Each patient had been diagnosed by at least two trained psychiatrists according to the criteria of the *Diagnostic and Statistical Manual of Mental Disorders, Fourth Edition* (*DSM-IV*) based on the Structured Clinical Interview for *DSM-IV* (SCID). The patients were excluded if they had neurological or medical conditions that might affect the central nervous system, such as atypical headaches, head trauma with loss of consciousness, chronic lung disease, kidney disease, chronic hepatic disease, thyroid disease, active cancer, cerebrovascular disease, epilepsy, seizures, substance-related disorders, or mental retardation, as previously described (32, 35–40). Patients were assessed at T1 and approximately 1.5 years later (T2) (mean duration between T1 and T2 = 1.71 ± 0.79 years). Among the patients, 61 received antipsychotics (1 who was typical, 53 who were atypical, and 7 who were a combination of typical and atypical), while 4 patients did not receive antipsychotics at T1. Chlorpromazine equivalents (CPZ-eqs.) and biperiden equivalents (Biperiden-eqs.) of taking a daily dose of total antipsychotics and anti-Parkinsonian drugs were calculated at T1 and T2, respectively (41). The clinical symptoms and side effects of the antipsychotics on the patients were evaluated using the Positive and Negative Syndrome Scale (PANSS) (42) and the Drug-Induced Extra-Pyramidal Symptoms Scale (DIEPSS) (43), respectively.

Written informed consent was obtained from all participants after the procedures had been thoroughly explained. This study was performed in accordance with the Declaration of Helsinki from the World Medical Association and was approved by the Research Ethics Committee of Osaka University.

### Measurement of Intellectual Function

We administered the Japanese version of the short form (44–46) of the Wechsler Adult Intelligence Scale (WAIS)-III (47) (WAIS-SF) to measure the IQ at T1 or T2 of patients with schizophrenia. The WAIS-SF consists of a dyad of similarities of verbal intellectual ability and symbol search of performance intellectual ability to determine the subject’s intellectual ability. The WAIS-SF is designed to be sensitive to functional outcome measures (daily living skills and social functioning). The WAIS-SF requires approximately 10 min to administer. A decrease in intelligence is assessed by comparing standard assessments of estimated premorbid IQ and IQ at T1 or T2 using the Japanese version of the National Adult Reading Test (JART) (48) and the WAIS-SF, respectively (44–46).

### Measurement of Daily Living Skills

The Japanese version of the UCSD Performance-Based Skills Assessment-Brief (UPSA-B) was administered to the patients to assess daily living skills (functional capacity) (44, 49–51). The UPSA-B assesses daily living activities through role-playing. The UPSA-B consists of two domains: i) financial skills (e.g., counting money and paying bills) and ii) communication skills (e.g., calling emergency services or rescheduling an appointment by telephone). Each domain score was converted into standard scores ranging from 0 to 50 points, and the total score was the sum of the standard scores, ranging from 0 to 100 points. A higher score indicates a higher level of daily living skills.

### Measurement of Social Functioning

The Japanese version of the SFS was administered to measure social functioning (20, 44, 52–54). The SFS has seven subscales: i) withdrawal (time spent alone, initiation of conversation, and social avoidance): the score ranges from 0 to 15 points; ii) interpersonal communication (number of friends/having a romantic partner and quality of communication): the score ranges from 0 to 12 points; iii) independence–performance (performance of skills necessary for independent living): the score ranges from 0 to 39 points; iv) independence–competence (ability to perform skills necessary for independent living): the score ranges from 0 to 39 points; v) recreation (engagement in a range of common hobbies, interests, pastimes, etc.): the score ranges from 0 to 45 points; vi) prosocial activities (engagement in a range of common social activities, e.g., sport): the score ranges from 0 to 66 points; and vii) employment/occupation (engagement in productive employment or a structured program with daily activities): the score ranges from 0 to 10 points. High internal consistency of the SFS has been represented by calculating Cronbach’s α for the SFS full scale (α > 0.80) and six subscales (i)–(vi) (ငα = 0.59–0.88), and mean item–total correlation coefficients for the SFS full scale (*r* = 0.56–0.71) and six subscales (i)–(vi) (*r* = 0.23–0.58) (20, 55). Subscale (vii) contains a filter item (employment yes/no) with different subsequent items; therefore, measures of the internal consistency have not been assessed (20, 55). Bivariate correlation coefficients between SFS full scale score and the seven subscale scores were high (*r* > 0.63), but the inter-correlation patterns for the three subscales (ii), (iv), and (vii) were somewhat lower (20, 55). Principal component analysis for patients with schizophrenia has revealed that two factors consisted of subscales (i)–(vi) and subscale (vii) solely (56). Furthermore, since the employment/occupation subscale (vii) is strongly correlated with social activity, subscale (vii) was excluded from this study. Therefore, the total score was the sum of the remaining six subscale scores (i)–(vi): scores range from 0 to 216 points. A higher score indicates a higher level of social functioning.

### Measurement of Social Activity

The SAA was administered to assess social activity as a measure of work outcomes. The SAA was developed based on the Modified Social Adjustment Scale—Work Outcome (57) and its Japanese version (58). The usefulness of the prototype of SAA, i.e., Modified Social Adjustment Scale (Work Outcome)—Japanese version, has been reported in a previous study (9). The SAA is divided into the following sections: work for pay, housework, and studying. In each section, the average work hours per week (h/week) were calculated from all work hours per week and the total number of weeks worked in the past 3 months. If subjects were eligible for more than one section, the average work hours per week (h/week) were added across three sections. The SAA was performed in an interview conducted by psychologists or psychiatrists.

### Statistical Analysis

All statistical analyses were performed using IBM SPSS Statistics 24.0 software (IBM Japan, Tokyo, Japan). Differences in demographic variables between assessments (T1 and T2) were analyzed using a paired t-test. Standardized effect sizes were calculated using Cohen’s d method (http://www.uccs.edu/faculty/lbecker). Each effect of IQ, daily living skills, social function, and/or clinical variables (T1, T2, or T2 − T1) on social activity (T1, T2, or T2 − T1) was analyzed using a linear regression model with social activity (T1, T2, or T2 − T1) as the dependent variable; IQ, daily living skills, social function, and/or clinical variables (T1, T2, or T2 − T1) as independent variables; and age and gender as covariates. Finally, stepwise forward multiple linear regression analyses were used for determining effects of these longitudinal changes in IQ, daily living skills, social function, and clinical variables on the longitudinal changes in social activity. The significance level was set at a two-tailed p < 0.05 for all statistical tests.

## Results

### Differences in IQ, Daily Living Skills, Social Function, and Social Activity Over the 1.5-Year Follow-Up Period

**Figure 1:**
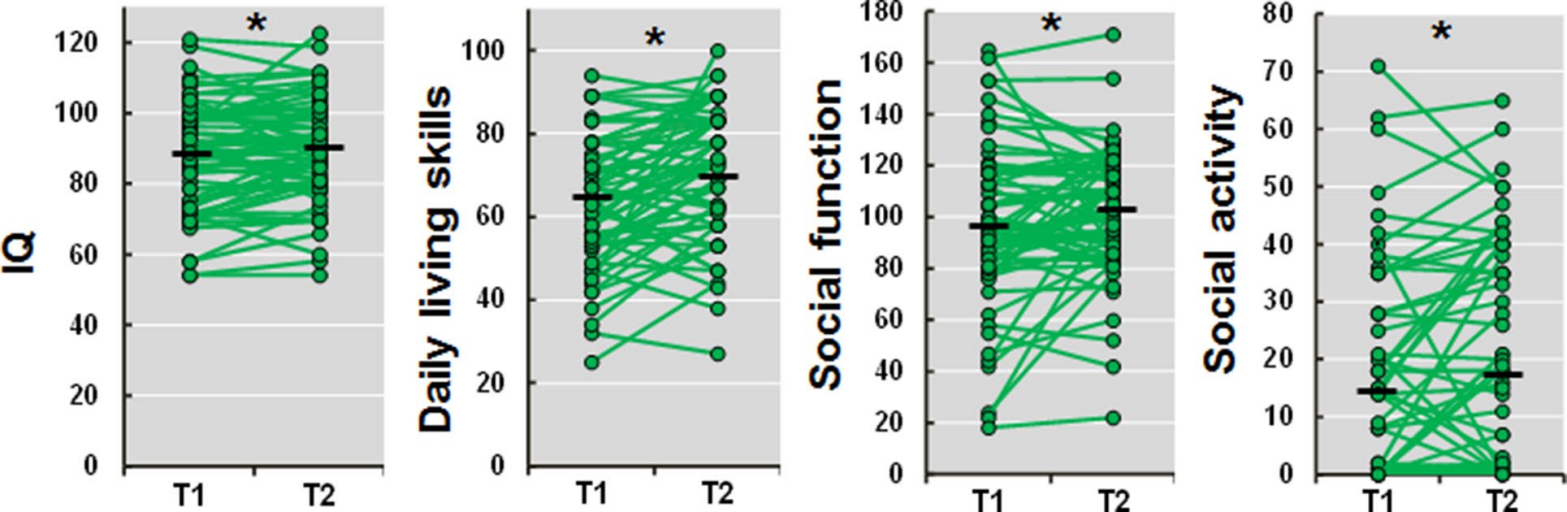
Longitudinal changes in IQ, daily living skills, social function, and social activity in patients with schizophrenia. T1, time 1 (baseline); T2, time 2 (follow-up). A higher score indicates a higher level of IQ, daily living skills, social function, and social activity, respectively. *p < 0.05.

The demographic information at the assessment points (T1 and T2) is shown in **Table 1**. The age and duration of illness at T2 were naturally greater than the values recorded at T1 (both *t* = 12.4, *p* = 1.32 × 10^−18^). The Biperiden-eqs. at T2 were significantly decreased compared with those at T1 (*t* = −3.2, *p* = 2.20 × 10^−3^), while the CPZ-eqs. were not significantly different between T1 and T2 (*t* = 1.6, *p* = 0.10). The positive and negative symptoms were significantly improved at T2 (positive, *t* = −2.2, *p* = 0.032; negative, *t* = −2.4, *p* = 0.019). We investigated 1.5-year longitudinal changes in IQ, daily living skills, social function, and social activity in patients with schizophrenia. All of these impairments differed significantly between assessments (T1 and T2) (**Figure 1**; IQ, *t* = 2.0, *p* = 0.048; daily living skills, *t* = 4.4, *p* = 3.60 × 10^−5^; social function, *t* = 2.1, *p* = 0.038; social activity, *t* = 2.0, *p* = 0.049). These impairments were improved at T2 compared with T1. However, the effect sizes of the longitudinal changes were small (Cohen’s *d* = 0.14–0.45).

**Table 1:** Demographic variables in 65 patients with schizophrenia at each assessment.

Variables	Baseline (T1)	Follow-up (T2)	Cohen’s d	*t*	*p*-values
**Gender (male/female)**	29/36	–	–	–	–
**Age at onset (years)**	23.3 ± 11.7	–	–	–	–
**Estimated premorbid IQ**	101.3 ± 10.1	–	–	–	–
**Education (years)**	13.5 ± 2.4	–	–	–	–
**Age (years)**	33.7 ± 12.8	35.5 ± 13.0	0.14	12.4	**1.32 × 10** **^−18^**
**Duration of illness (years)**	10.4 ± 9.1	12.2 ± 9.2	0.20	12.4	**1.32 × 10** **^−18^**
**CPZ-eq. total (mg/day)**	685.3 ± 562.4	748.3 ± 638.9	0.10	1.6	0.10
**CPZ-eq. atypical (mg/day)**	673.6 ± 563.4	737.7 ± 610.8	0.11	1.7	0.09
**CPZ-eq. typical (mg/day)**	11.8 ± 50.4	10.6 ± 52.6	−0.02	−0.1	0.90
**Biperiden-eq. (mg/day)**	1.1 ± 1.9	0.4 ± 0.9	−0.47	−3.2	**2.20 × 10** **^−3^**
**Positive symptoms**	18.0 ± 5.5	16.5 ± 5.6	−0.27	−2.2	**0.032**
**Negative symptoms**	19.9 ± 5.6	18.6 ± 5.0	−0.24	−2.4	**0.019**
**IQ**	87.9 ± 15.3	90.0 ± 14.3	0.14	2.0	**0.048**
**Intelligence decline**	−13.5 ± 13.8	−11.4 ± 13.1	0.16	2.0	**0.048**
**Daily living skills**	64.2 ± 15.6	71.2 ± 15.6	0.45	4.4	**3.60 × 10** **^−5^**
**Social function**	96.4 ± 31.4	102.7 ± 24.7	0.22	2.1	**0.038**
**Social activity (h/week)**	14.7 ± 18.2	17.8 ± 19.5	0.16	2.0	**0.049**

T1, time 1; T2, time 2; IQ, intelligence quotient; CPZ-eq., chlorpromazine equivalents of total antipsychotics. Means ± SD are shown. Complete demographic information was not obtained for all subjects (positive and negative symptoms, n = 63). P-values < 0.05 are shown in boldface and are underlined.

### Effects of the IQ, Daily Living Skills, and Social Function on Social Activity

**Figure 2:**
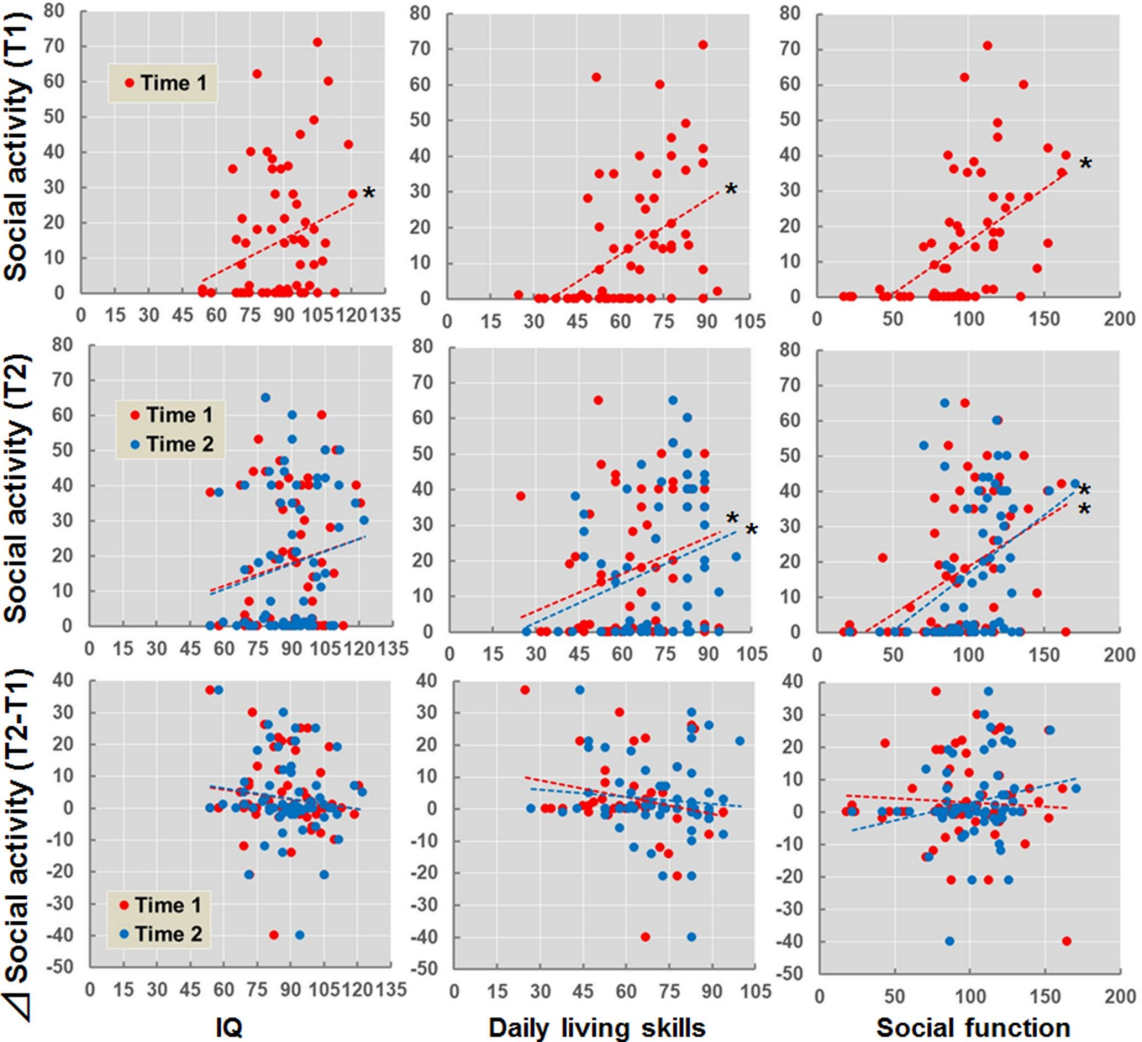
Effects of IQ, daily living skills, and social function at T1 and T2 on social activity (T1, T2, and T2 − T1) in patients with schizophrenia. *p < 0.05. Δsocial activity (T2 − T1) indicates subtraction of social activity at T1 from social activity at T2.

The estimated longitudinal trajectory of social activity in patients with schizophrenia is shown in **Supplementary Figure 1**. The social activities recorded at T1 or T2 were not associated with the duration of the illness (*p* > 0.05). We examined the effects of IQ, daily living skills, and social function recorded at T1 and T2 on social activity (T1, T2, and T2 − T1) (**Figure 2**). IQ (*beta* = 0.28, *p* = 0.030), daily living skills (*beta* = 0.43, *p* = 3.91 × 10^−4^), and social function (*beta* = 0.52, *p* = 1.27 × 10^−5^) recorded at T1 were positively correlated with social activity at T1. Additionally, daily living skills and social function recorded at both T1 (daily living skills, *beta* = 0.28, *p* = 0.026; social function, *beta* = 0.43, *p* = 3.95 × 10^−4^) and T2 (daily living skills, *beta* = 0.31, *p* = 0.014; social function, *beta* = 0.42, *p* = 8.83 × 10^−4^) were also positively correlated with social activity at T2, although correlations between IQ and social activity were marginal (T1, *beta* = 0.21, *p* = 0.10; T2, *beta* = 0.25, *p* = 0.067). In contrast, correlations were not observed between IQ, daily living skills, or social function recorded at T1 and T2 and the longitudinal changes in social activity (T2 − T1) (*p* > 0.05).

### Effects of Clinical Variables on Social Activity

We further examined the potential effects of clinical variables such as psychiatric symptoms and antipsychotics recorded at T1 and T2 on social activity (T1, T2, and T2 − T1) (**Supplementary Figure 2**). The positive (*beta* = −0.30, *p* = 0.016) and negative (*beta* = −0.32, *p* = 0.011) symptoms recorded at T1 were negatively correlated with social activity at T1. The positive and negative symptoms recorded at both T1 (positive, *beta* = −0.35, *p* = 6.01 × 10^−3^; negative, *beta* = −0.33, *p* = 0.010) and T2 (positive, *beta* = −0.60, *p* = 2.95 × 10^−7^; negative, *beta* = −0.66, *p* = 3.26 × 10^−9^) were also negatively correlated with social activity at T2. In terms of medications, the CPZ-eqs. or Biperiden-eqs. recorded at the two assessments were not significantly correlated with social activity (*p* > 0.05), with the exception of CPZ-eq. at T2 (*beta* = −0.28, *p* = 0.031). Furthermore, correlations were not observed between these clinical variables at T1 and T2 and the longitudinal changes in social activity (T2 −T1) (*p* > 0.05).

### Relationships Between Longitudinal Changes in IQ, Daily Living Skills, Social Function, and Clinical Variables With Longitudinal Changes in Social Activity

**Figure 3:**
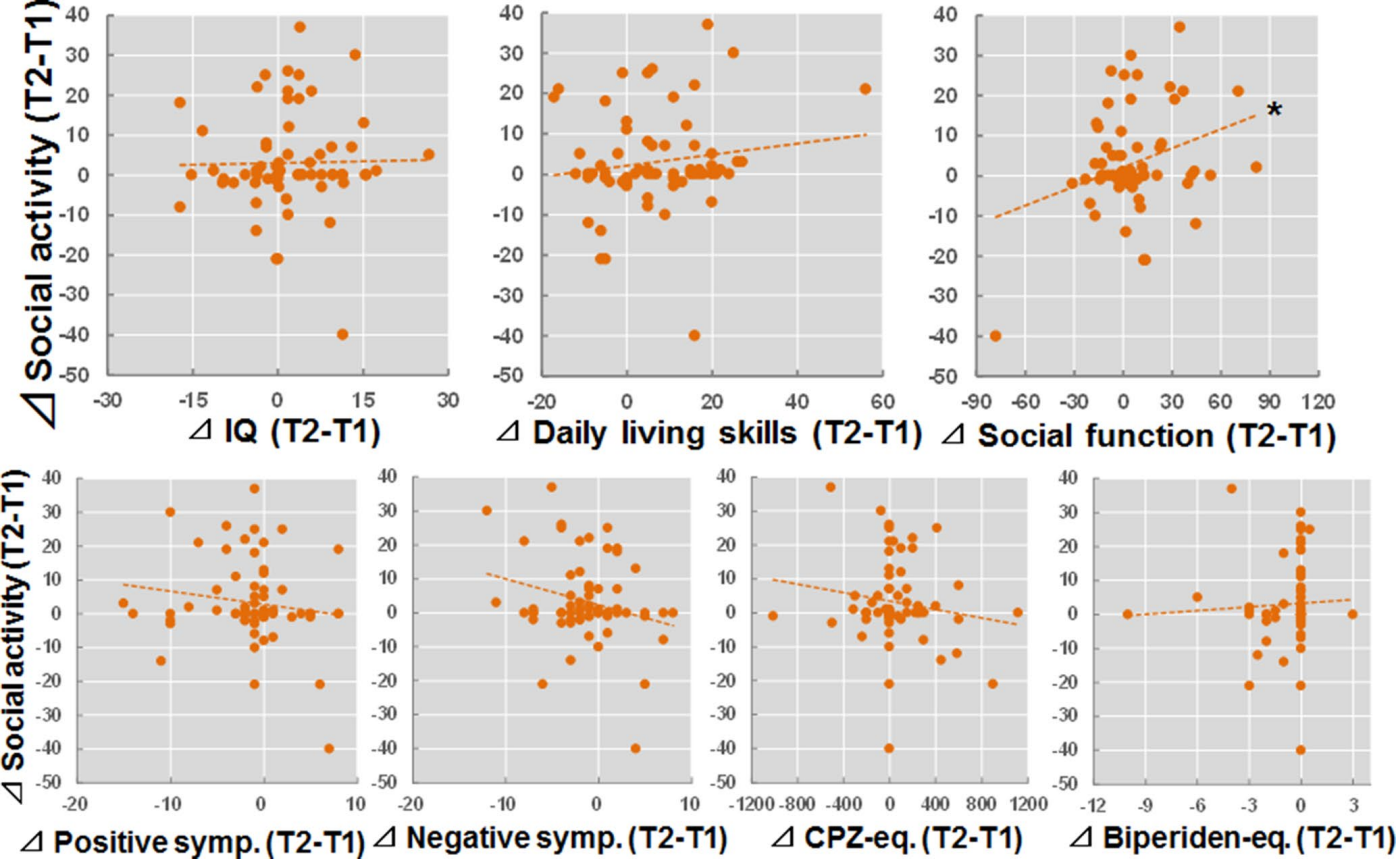
Relationships between longitudinal changes in IQ, daily living skills, social function, and clinical variables (T2 − T1) and changes in social activity (T2 − T1) in patients with schizophrenia. *p < 0.05.

We investigated the relationships between longitudinal changes in IQ, daily living skills, social function, and clinical variables (T2 − T1) and longitudinal changes in social activity (T2 − T1) in patients with schizophrenia (**Figure 3**). The longitudinal change in social function was positively correlated with a change in social activity (*beta* = 0.26, *p* = 0.036). In contrast, significant correlations were not observed between longitudinal changes in IQ, daily living skills, or clinical variables with longitudinal changes in social activity (*p* > 0.05).

To determine which independent variables showed significant predictors for the longitudinal change in social activity, the longitudinal changes in IQ, daily living skills, social function, and clinical variables were included in a stepwise regression model. In the analysis, the longitudinal change in social function was independently correlated with the longitudinal change in social activity (**Table 2**, Model 1; adjusted *R* *^2^* = 0.11, *F* = 8.64, *p* = 4.63 × 10^−3^). The longitudinal changes in other factors did not directly affect the change in social activity (*p* > 0.05). We further investigated which subscale of social function was strongly correlated with longitudinal changes in social activity using a stepwise regression model. Of the six subscales of social function, longitudinal changes in interpersonal communication were positively correlated with changes in social activity (Model 2; adjusted *R* *^2^* = 0.11, *F* = 8.77, *p* = 4.32 × 10^−3^).

**Table 2:** Stepwise multiple regression analyses for longitudinal changes in social activity (T2 − T1) in patients with schizophrenia.

Independent variables	Change (T2 − T1)	T1	T2	B	SE	*beta*	*p-values*
**Model 1;** ***R*** ***^2^*** **= 0.11,** ***F*** **= 8.64,** ***p*** **= 4.63 × 10** **^−3^**							
⊿** Total score of social function**	6.3 ± 24.1	96.4 ± 31.4	102.7 ± 24.7	0.18	0.06	0.35	**4.63 × 10** **^−3^**
**Model 2; ** ***R*** ***^2^*** **= 0.11, ** ***F*** **= 8.77, ** ***p*** **= 4.32 × 10** **^−3^**							
⊿** Interpersonal communication**	0.4 ± 2.5	6.6 ± 2.8	7.0 ± 2.7	1.72	0.58	0.35	**4.32 × 10** **^−3^**

T1, time 1; T2, time 2; SE, standard error. Means ± SD are shown. P-values < 0.05 are shown in boldface and underlined. Probability of F to enter ≤0.05, probability of F to remove ≥0.10.

## Discussion

In this study, we evaluated the contributions of cognitive function, daily living skills, social function, illness severity, and medications to social activity in patients with schizophrenia. This study is the first to systematically investigate the effects of longitudinal changes in these factors on longitudinal changes in social activity in patients with schizophrenia. The follow-up duration between assessments was approximately 1.5 years. The longitudinal change in social activity was positively correlated with the longitudinal changes in social function, particularly interpersonal communication. Improvements in interpersonal communication may be a useful indicator of recovery in clinical trials of patients with schizophrenia.

Consistent with the results from previous studies (1, 59), negative symptoms exerted a strong effect on social activity in patients with schizophrenia in the present study. Negative symptoms correlated with real-world functioning in many studies. Negative symptoms, such as apathy, are critical in predicting functional outcomes (4, 60, 61). We further examined possible correlations among longitudinal changes in social activity, cognitive functions, daily living skills, social function, illness severity, and medications (**Supplementary Table 1**). Improvements in negative symptoms were associated with the improvements in daily living skills (*beta*=−0.27, *p* = 0.038) of these factors. However, the longitudinal changes in social activity were affected by the change in social function (*beta* = 0.26, *p* = 0.036) but not negative symptoms (*beta*=−0.21, *p* = 0.09). The longitudinal relationship between social activity and social function remained significant, even after considering negative symptoms (**Table 2**). Compared with improvements in negative symptoms, improvements in social function may be a more sensitive marker for improvements in social activity.

Of the 65 patients with schizophrenia, 25 patients (38.5%) were unemployed, homemakers, or students at the time of the T1 assessment, corresponding to 22 patients (33.8%) at T2. Our participants included a mixed cohort of patients who were in the early and chronic phases of schizophrenia at T1. The duration of illness at T1 was 10.4 ± 9.1 years, ranging from 0 to 40 years. Approximately 60% of patients had been affected by schizophrenia for >5 years. Our participants are considered as being in a more chronic and symptomatic stable phase of the illness. Similar to chronic-phase patients, first-episode patients display impairments in cognitive function, daily living skills, social function, and social activity (2, 62, 63). On the other hand, we did not observe a correlation between social activity and the duration of the illness (*p* > 0.05) or a difference in social activity between patients with short (<5 years) and those with long (>5 years) illness durations (*p* > 0.05). Based on these findings, social activity is not affected by the duration of illness in our patients.

The patients in this study did not receive any specialized interventions for social activity over the 1.5-year follow-up period. The current study was not a part of a larger clinical intervention trial. When patients are enrolled in structured programs and seeking employment, higher baseline cognitive function and participation in cognitive remediation interventions contribute to vocational success (12, 64, 65). Accumulating evidence supports the efficacy of social cognitive interventions for patients with schizophrenia (66). Despite the absence of any social activity intervention in our participants, social activity improved over the follow-up period. However, we have held several workshops on a brief assessment of the decrease in intelligence in patients with schizophrenia to promote the concept of monitoring the decrease in intelligence of Japanese patients with schizophrenia (46). In addition, we have recommended that physicians should prescribe only atypical antipsychotics without anticholinergics to reduce the cognitive impairments observed in patients with schizophrenia (46). Therefore, Biperiden-eq. at T2 (0.4 ± 0.9) decreased significantly compared to T1 (1.1 ± 1.9). As mentioned in the Methods section, 61 patients received antipsychotics (1 who was typical, 53 who were atypical, and 7 who were a combination of typical and atypical), while 4 patients did not receive antipsychotics at T1. At T2, 64 patients received antipsychotics (1 typical, 59 atypical, and 4 a combination of typical and atypical), while only one patient did not receive antipsychotics. Although the longitudinal changes in these medications were not directly correlated with the changes in social activity, the changes in these medications might have indirectly contributed to the changes in social activity.

Consistent with findings from previous studies (9, 67), significant cross-sectional associations were observed among IQ, daily living skills, social function, psychiatric symptoms, and social activity at T1 and T2 in patients with schizophrenia. Daily living skills, social function, and psychiatric symptoms at the T1 baseline were prospectively associated with social activity at the T2 follow-up. Although relationships were not observed between the levels of these factors at T1 and T2 and changes in social activity, our 1.5-year longitudinal associations revealed that longitudinal change in social function, particularly interpersonal communication, significantly affected longitudinal aspects of social activity. Consistent with previous studies (68), negative symptoms had greater impact on social function than positive symptoms (*p* < 0.05). Furthermore, the scores on interpersonal communication recorded at both T1 and T2 were negatively correlated with positive and negative symptom scores at T1 (positive, *beta* = −0.36, *p* = 4.14 × 10^−3^; negative, *beta* = 0.41, *p* = 9.14 × 10^−4^) and T2 (positive, *beta* = −0.28, *p* = 0.028; negative, *beta* = −0.44, *p* = 2.86 × 10^−4^), respectively. In contrast, longitudinal changes in interpersonal communication were not significantly correlated with changes in any psychiatric symptoms (*p* > 0.05). Thus, improvements in social function, particularly interpersonal communication, are a valuable indicator of long-term work outcomes and may be an important treatment target in patients with schizophrenia that could lead to improvements in social activity. Further research is needed to obtain a better understanding of the determinants of social activity to identify a novel therapeutic target for schizophrenia. Integrated and personalized programs aimed at improving interpersonal communication and social activity may be provided as a standard treatment to patients with schizophrenia.

Some limitations exist regarding the interpretation of our findings. Our participants consisted of inpatients (*n* = 10) and outpatients (*n* = 55) at the baseline assessment. The hospitalization status might affect our findings. However, longitudinal differences in social activity were not observed between inpatients (*t* = −0.75, *p* = 0.47) and outpatients (*t* = −1.86, *p* = 0.069) (**Supplementary Figure 3**). In addition, the relationship between longitudinal changes in social function and social activity was still significant even after adjusting for the hospitalization status (*p* < 0.05). This study is limited by its small sample size (*n* = 65) and by the absence of data regarding the effects of these factors on social activity in healthy subjects over a 1.5-year period. Further studies using a larger sample including healthy subjects are needed to verify our findings. Because social activity is highly correlated with IQ, daily living skills, social function, and psychiatric symptoms and they are correlated to each other, these correlations are not independent. Therefore, we did not apply Bonferroni correction for multiple comparisons in this study. This could lead to a type I error. Our results should be interpreted with caution. As mentioned in *Methods*, the employment/occupation subscale (vii) of the SFS was excluded from this study. Regarding the research fields of social activity, we recommend using the six-subscale version (i)–(vi) of the SFS to assess social activity. However, further study is needed to validate the six-subscale version of the SFS. The social activity assessment performed in this study was a quantitative measure of time (h/week) and not a subjective measure of the work quality. The quality of the time spent in social activity may be more important than the quantity of social activity.

In conclusion, IQ, daily living skills, social function, and social activity were nominally improved over a 1.5-year follow-up period in patients with schizophrenia. Baseline and follow-up IQ, daily living skills, and social function were positively correlated with social activity at both the baseline and 1.5-year follow-up assessments, suggesting that cognitive and social functions might be cross-sectionally and prospectively related to social activity. A long-term change in social activity was affected by longitudinal changes in social function, particularly interpersonal communication. Therefore, we postulate that the long-term decrease in social activity might be attenuated or improved by interventions targeting interpersonal communication skills.

## Ethics Statement

Written informed consent was obtained from all participants after the procedures had been thoroughly explained. This study was performed in accordance with the Declaration of Helsinki from the World Medical Association and was approved by the Research Ethics Committee of Osaka University.

## Author Contributions

KO was critically involved in the study design, analyzed and interpreted the data, and wrote the manuscript. RH supervised the entire project, was critically involved in the study design, data analysis, and interpretation, and was responsible for performing the literature review. CS, HF, YY, HY, MF, and TS were involved in subject recruitment and the clinical diagnostic assessments and contributed intellectually to the data interpretation. All authors contributed to and approved the final manuscript.

## Funding

This work was supported by Grants-in-Aid for Scientific Research (B) (25293250 and 16H05375), Scientific Research (C) (19K08081), and Young Scientists (B) (16K19784) from the Japan Society for the Promotion of Science (JSPS); the Health and Labour Sciences Research Grants for Comprehensive Research on Persons with Disabilities from the Japan Agency for Medical Research and Development (AMED); and a grant for Brain Mapping by Integrated Neurotechnologies for Disease Studies (Brain/MINDS) (AMED).

## Conflict of Interest Statement

The authors declare that the research was conducted in the absence of any commercial or financial relationships that could be construed as a potential conflict of interest.
