# Supplementary material for: A 1.5-Year Longitudinal Study of Social Activity in Patients With Schizophrenia
Source: Front Psychiatry. 2019 Aug 9;10:567. doi: 10.3389/fpsyt.2019.00567 (PMC6697059; doi:10.3389/fpsyt.2019.00567)
Supplement: Supplementary file 1 [file DataSheet_1.doc]

**Supplementary Information**


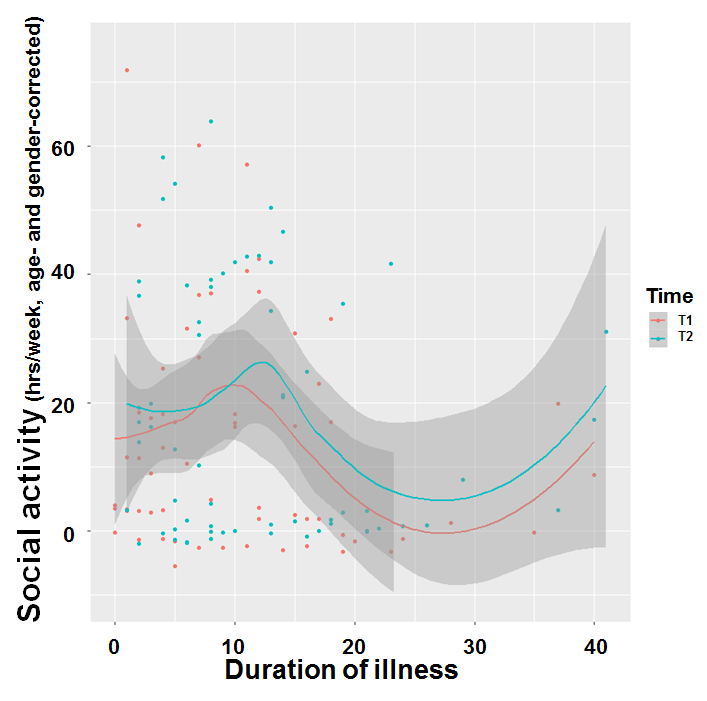


**Supplementary Fig. 1** **Estimated longitudinal trajectory of social activity in patients with schizophrenia**. Age- and gender-corrected social activity in each patient is shown as a dot (red=T1, blue=T2). Colored lines (red=T1, blue=T2) represent a LOESS fit across the duration of illness.


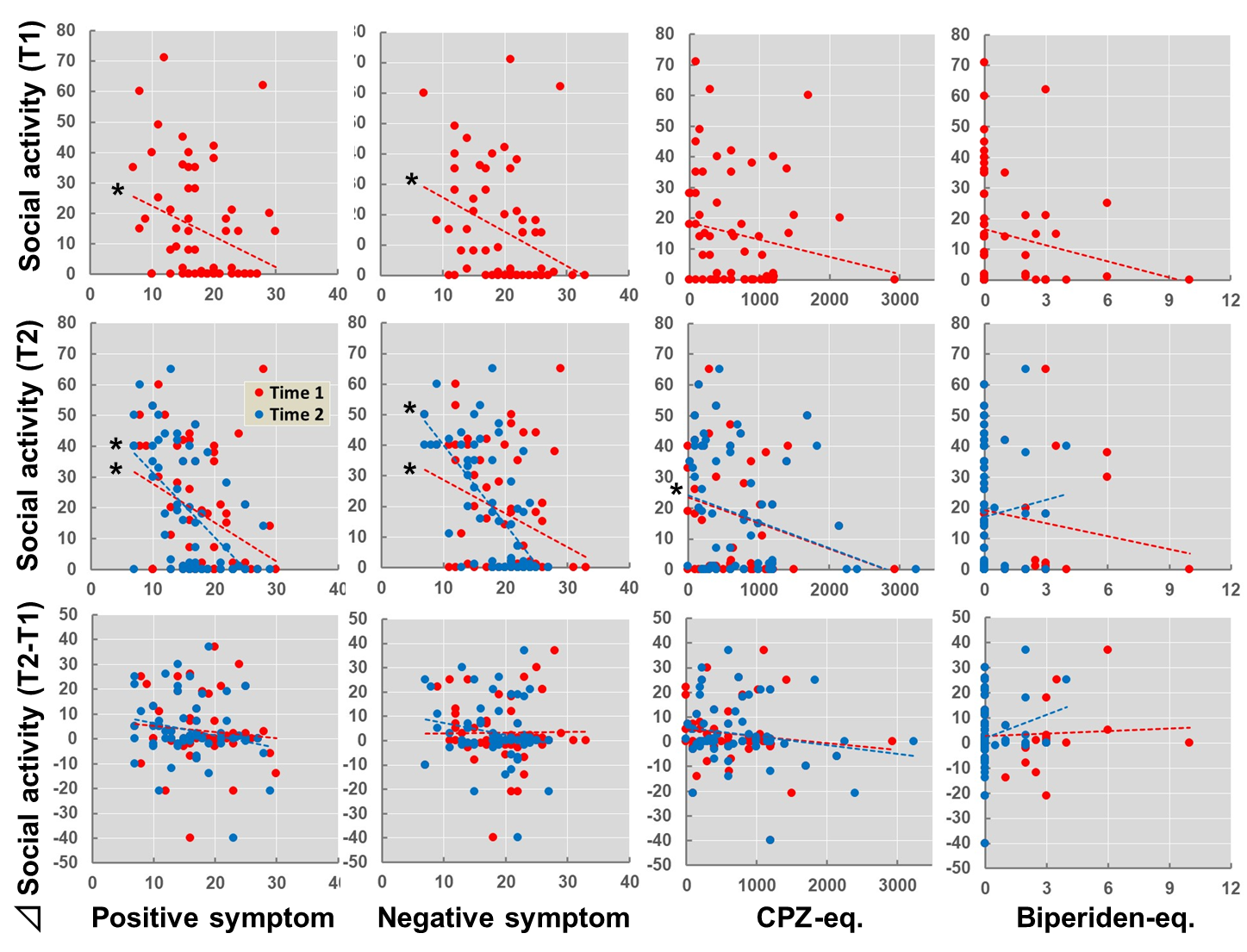


**Supplementary Fig. 2** **Effects of clinical variables recorded at T1 and T2 on social activity (T1, T2 and T2-T1) in patients with schizophrenia**. CPZ-eq.; chlorpromazine equivalents of total antipsychotics. **p*<0.05.


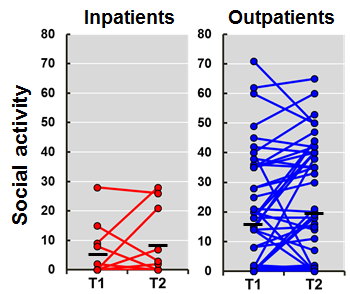


**Supplementary Fig. 3** **Longitudinal changes in social activity in inpatients or outpatients with schizophrenia at T1**. Longitudinal differences in social activity were not observed among inpatients (*n*=10, *t*=0.75, *p*=0.47) or outpatients (*n*=55, *t*=1.86, *p*=0.069). T1, rime 1 (baseline); T2, time 2 (follow-up).

**Supplementary Table 1 Correlations among longitudinal changes in social activity, cognitive functions, daily living skills, social function, illness severity and medications.**

|  |  | **1** | **2** | **3** | **4** | **5** | **6** | **7** | **8** |
| --- | --- | --- | --- | --- | --- | --- | --- | --- | --- |
| **1** | **⊿ Social activity** | - | 0.21 | 0.16 | **0.26*** | -0.14 | -0.21 | -0.15 | 0.04 |
| **2** | **⊿ IQ** | 0.21 | - | 0.14 | 0.08 | 0.01 | -0.15 | **-0.26*** | -0.17 |
| **3** | **⊿ Daily living skills** | 0.16 | 0.14 | - | -0.03 | **-0.31*** | **-0.27*** | -0.08 | -0.13 |
| **4** | **⊿ Social function** | **0.26*** | 0.08 | -0.03 | - | -0.13 | -0.17 | 0.07 | -0.20 |
| **5** | **⊿ Positive symptoms** | -0.14 | 0.01 | **-0.31*** | -0.13 | - | **0.76***** | 0.07 | 0.17 |
| **6** | **⊿ Negative symptoms** | -0.21 | -0.15 | **-0.27*** | -0.17 | **0.76***** | - | 0.22 | 0.04 |
| **7** | **⊿ CPZ-eq.** | -0.15 | **-0.26*** | -0.08 | 0.07 | 0.07 | 0.22 | - | -0.03 |
| **8** | **⊿ Biperiden-eq.** | 0.04 | -0.17 | -0.13 | -0.20 | 0.17 | 0.04 | -0.03 | - |

These correlations were analyzed using a linear regression model with age and gender as covariates. *Beta* is shown. *P*-values<0.05 are shown in boldface and underlined. **p*<0.05, ***p*<0.01 and ****p*<0.001.
